# Split-thickness skin graft using scalp as a donor site for third-degree burn treatment in a newborn (case report)

**DOI:** 10.1016/j.ijscr.2025.111897

**Published:** 2025-09-04

**Authors:** Paula Andrea Pérez Franco, Sebastian Murcia Espino, Mauricio Alfonso Uribe Rodríguez, Juan Felipe Vera Rodríguez, Jorge Elias Ochoa Martinez

**Affiliations:** a Plastic Surgeon - Universidad Militar Nueva Granada, Colombia; b Epidemiology Specialization Program, Universidad Autónoma de Bucaramanga, Bucaramanga, Colombia; c Postgraduate Student, Epidemiology Specialization Program, Universidad Autónoma de Bucaramanga, Bucaramanga, Colombia; d Medical doctor - Universidad El Bosque, Bogotá, Colombia

**Keywords:** Burns, Neonatal burns. Partial-thickness skin graft, Scalp skin graft, Homologous skin graft, Cadaveric skin allograft

## Abstract

**Introduction and importance:**

Neonatal burns are uncommon but represent a critical medical challenge due to the physiological vulnerability of newborns, including fragile skin, immature immune response, and limited physiological reserves. Most burns in this population are iatrogenic, often preventable with proper care and equipment handling. The absence of standardized protocols complicates management, making each case an opportunity to refine treatment strategies and highlight prevention.

**Case presentation:**

We present a case of a 16-hour-old male newborn with a third-degree thermal contact burn to the right forearm caused by overheated saline bags. Initial treatment included autolytic debridement and temporary coverage with a cadaveric skin allograft. Definitive closure was achieved using a split-thickness autologous skin graft harvested from the scalp.

**Clinical discussion:**

The use of a cadaveric skin allograft improved wound bed conditions, facilitating successful autologous grafting. The scalp donor site provided rapid healing, minimal scarring, and early hair regrowth. This case reinforces the importance of careful thermal control in clinical environments and demonstrates the efficacy of a staged surgical approach in neonatal patients.

**Conclusion:**

Scalp skin grafting is a reliable option in neonates, offering favorable healing and cosmetic outcomes. A two-stage grafting strategy can enhance graft success and minimize donor site morbidity.

## Highlights

• Neonatal third-degree burn successfully managed with a scalp-derived STSG.
• Homologous grafts optimized wound bed, enabling effective autologous coverage.
• Emphasizes the need for strict temperature control of medical equipment and supplies near neonates.
• Case supports scalp as an optimal donor site for newborn burn reconstruction.
• Highlights importance of a stepwise, minimally invasive approach in neonatal burns

## 1 Introduction

Neonatal burns, although rare, represent a significant medical challenge due to the unique physiological characteristics of newborns and the profound impact on the patient, family, and healthcare system. The etiology of these injuries is diverse, with a notable distinction between iatrogenic and domestic causes. A systematic review of global literature identified 105 reported cases over four decades, emphasizing that contact burns are the most frequent, followed by flame burns and scalds. Importantly, nearly 60 % of these injuries occur in hospital settings, often resulting from human error, malfunctioning medical devices, or substandard infrastructure within healthcare institutions [1,2,4].

In developed countries, iatrogenic burns are more prevalent and tend to occur at an earlier age, often affecting neonates with lower birth weights compared to those with domestic burns. For instance, a study conducted in Romania reported that all burns in premature neonates were iatrogenic, resulting from a fire in a maternity hospital, whereas full-term neonates experienced fewer such injuries [1]. Moreover, the management of neonatal burns is inherently complex due to the fragility and thinness of the skin, immature immune responses, and the high risk of long-term complications and sequelae. These challenges underscore the necessity of a multidisciplinary approach [2].

The rarity and severity of neonatal burns necessitate specialized care and targeted prevention strategies. Establishing well-equipped burn centers and implementing comprehensive health education programs are essential steps toward reducing their incidence and improving clinical outcomes. This article presents a preventable injury that occurred during a medical procedure in a newborn within a healthcare facility, and describes the therapeutic approach and clinical considerations used to resolve the case based on current evidence [3,4].

In adults, partial-thickness skin graft donor sites commonly include the extremities and gluteal region. However, these areas are generally avoided in small children due to both anatomical and practical limitations. Pediatric limbs are thinner and smaller than those of adults, offering limited surface area for harvesting, while the gluteal region lies within the diaper area, thereby increasing the risk of contamination and complicating postoperative care. Moreover, donor sites in these locations are associated with disadvantages such as delayed healing, aesthetically unfavorable scarring, and the need to prepare a separate sterile surgical field, which can increase procedural complexity. Donor site pain is also a major concern, as frequent contact with clothing and movement may disrupt healing and impair comfort during daily activities [5,6].

Crawford first described the scalp as a donor site in 1964, pioneering its use in burn treatment. Since then, several plastic surgeons have adopted the scalp for skin grafting in burn patients [7]. The scalp offers multiple advantages as a donor site, including consistent reliability, a low complication rate, and rapid healing attributed to its rich vascular supply. Moreover, it allows for early hair regrowth and minimal visible scarring, facilitated by accelerated wound healing and the natural camouflage provided by hair [5]. The scalp is widely recognized as a suitable donor site in the pediatric population [8,9]; however, potential postoperative complications include hypertrophic scarring, folliculitis, and localized alopecia [10].

## 2 Methods

This case report has been conducted and documented in accordance with the SCARE66 (Surgical CAse REport) criteria, ensuring adherence to established methodological67standards for the transparent and complete reporting of surgical case reports. This68framework was followed to enhance the clarity, reproducibility, and academic value of the69presented clinical case [11].

## 3 Case presentation and results

A male newborn, born at 39 weeks of gestation and 16 h old at the time of injury, was admitted to our institution for evaluation by the burn unit and the plastic and reconstructive surgery service. The patient sustained a thermal contact burn at 16 h of life, caused by exposure to a hot solid object—specifically, heated saline solution bags of unknown temperature—applied to the right upper limb. The burn affected approximately 1 % of the total body surface area (TBSA).

**Fig. 1:**
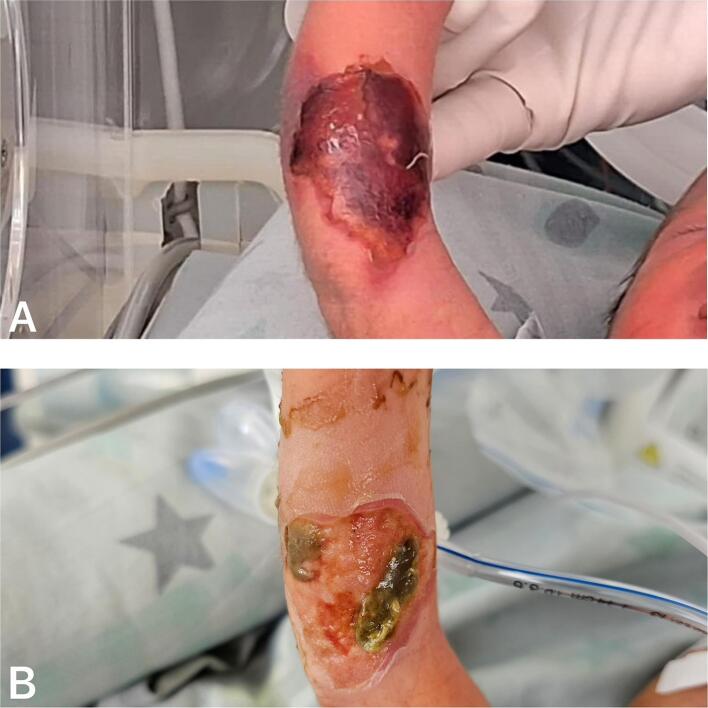
A. Third-degree contact burn affecting the proximal third of the forearm and the antecubital fold. B. Partial eschar removal following the application of hydrogel dressings for 7 days.

Upon admission to the tertiary hospital, the patient was evaluated by the burn unit and the plastic surgery team. The examination revealed a third-degree burn with areas of second-degree involvement, affecting the antecubital fold and the proximal third of the forearm in the radial palmar region (Fig. 1A). Hydrogel dressings were applied to initiate autolytic debridement and facilitate partial removal of the necrotic eschar (Fig. 1B).

## 4 Surgical procedures

Prior to any surgical procedure, prophylactic antibiotics were administered using intravenous cefazolin at a standard dose of 30 mg/kg, given 30 min before incision.

### 4.1 First surgical stage

**Fig. 2:**
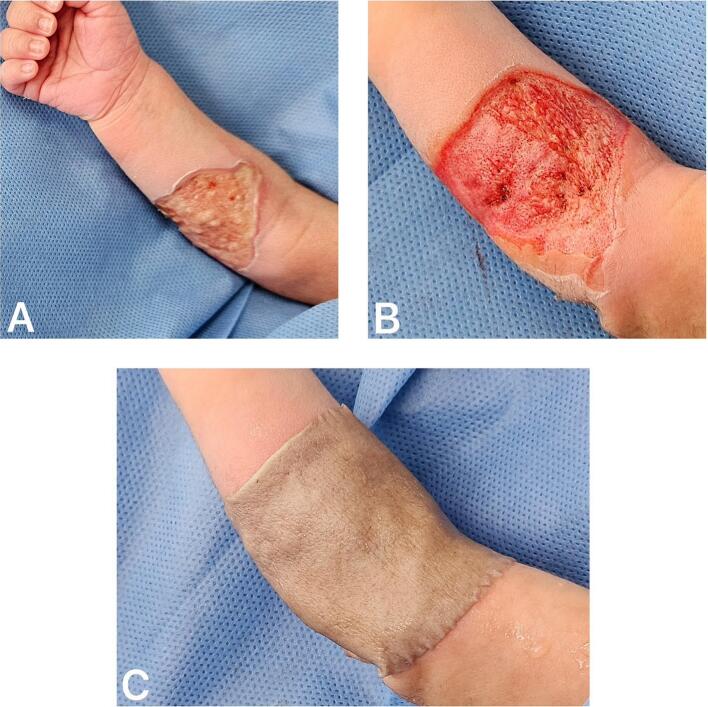
A. Wound bed exposure following surgical cleansing. B. Residual defect after escharectomy. C. Application of cadaveric skin allograft.

After removing the bandages and dressings, the wound was cleansed, revealing adherent whitish eschar (Fig. 2A). Escharectomy and debridement of necrotic eschar and devitalized tissue were performed (Fig. 2B). Minimal bleeding was observed and controlled using adrenaline-impregnated compresses and saline solution. A cadaveric skin allograft preserved in glycerol, obtained from the tissue bank, was applied to the wound bed after rinsing it with saline to remove residual glycerol (Fig. 2C). The graft was then covered with nitrofurazone-impregnated gauze and secured using secondary non-elastic bandages. The allograft was provided by the District Institute of Science, Biotechnology, and Health Innovation (IDCBIS, by its Spanish acronym).

### 4.2 Second surgical stage

**Fig. 3:**
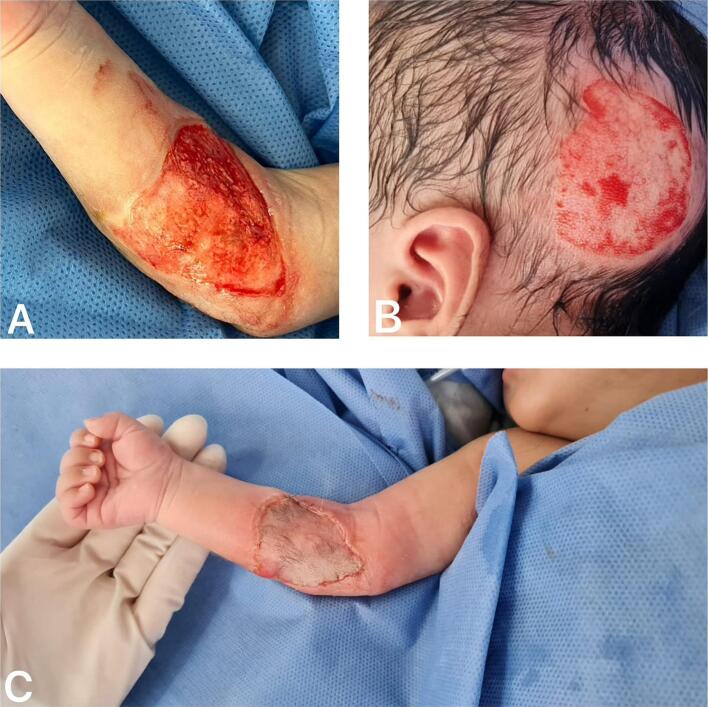
A. Surgical debridement following removal of the cadaveric skin allograft. B. Scalp as a donor site for autologous skin graft harvesting. C. Autologous skin graft placed over the radial-palmar forearm defect.

One week later, a second surgical procedure was performed. The previously applied cadaveric skin allograft from the tissue bank was removed, revealing hypertrophic granulation tissue. Surgical debridement was performed to level the wound bed to the plane of healthy tissue (Fig. 3A). Moderate bleeding was observed and was successfully controlled using saline- and adrenaline-impregnated compresses.

Once hemostasis was achieved, a partial-thickness skin graft was harvested from the left parietal scalp after shaving the area with a scalpel blade. Subdermal infiltration of the scalp was performed using 1000 mL of 0.9 % saline solution mixed with 1 mg of adrenaline to induce tumescence and facilitate uniform graft harvesting. A 0.2 mm-thick partial-thickness skin graft was then obtained using an electric dermatome (Padgett Model B). This motor-driven device lacks built-in pressure regulation; thus, the operator must apply consistent and uniform pressure manually during harvesting. The rotational speed depends on the motor used and typically ranges between 3000 and 5000 rpm (Fig. 3B).

Hemostasis at the donor site was achieved using adrenaline-impregnated compresses with saline, and the area was covered with petrolatum gauze. The harvested graft was then placed over the original radial-palmar forearm burn site (Fig. 3C) and secured with simple interrupted sutures using 5-0 Monocryl. Finally, the graft was covered with nitrofurazone-impregnated gauze and stabilized with secondary non-elastic bandages.

**Fig. 4:**
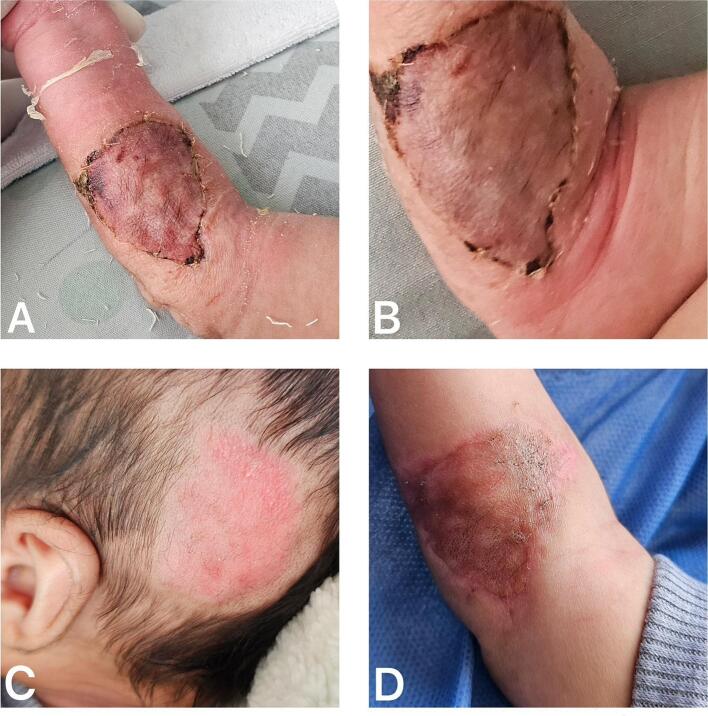
A. First dressing removal, 7 days after autologous graft placement with >95 % survival. B. Partial-thickness skin graft showing >95 % survival at 12 days postoperatively. C. Proper epithelialization of the donor site, along with early hair regrowth. D. Complete graft integration and epithelialization at 1-month follow-up.

At the time of dressing removal, seven days after placement of the autologous graft, the recipient site showed adequate integration of the partial-thickness skin graft, with more than 95 % graft survival. The area was cleansed using moist gauze soaked in saline solution and covered again with nitrofurazone-impregnated gauze and stabilized with secondary non-elastic bandages (Fig. 4A). At the donor site, the dressing was replaced, continuing with petrolatum gauze. Five days later, a second dressing change revealed complete graft integration (Fig. 4B). At one month postoperatively, the patient was reassessed during a follow-up visit, demonstrating full graft integration and proper epithelialization of the donor site, along with early hair regrowth (Fig. 4C, D).

**Fig. 5:**
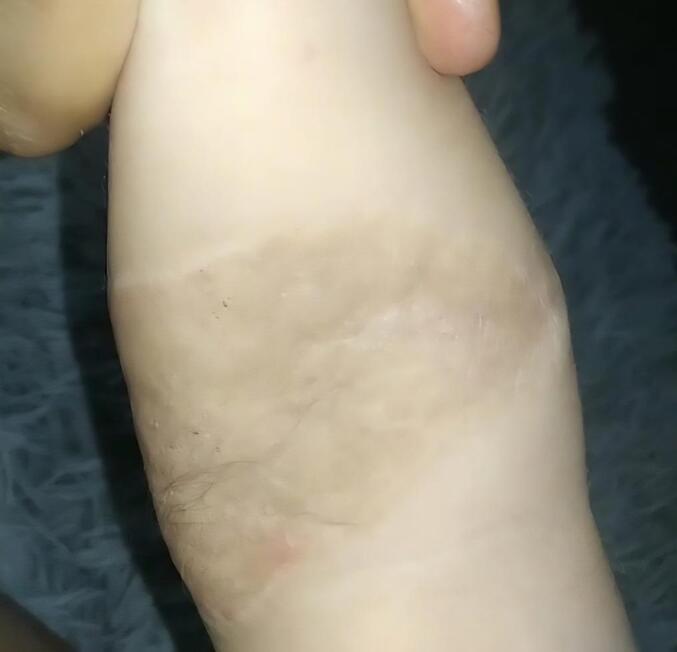
Photograph provided by the patient's mother, showing adequate graft integration with no contracture or retraction that could compromise the patient's mobility.

A formal 12-month in-person follow-up was not possible due to the patient's residence in a remote rural area, which presents significant transportation challenges for the family. However, a telemedicine consultation was conducted with the patient's mother, during which she provided updated clinical information and several photographs of the graft and donor site. The image with the best quality was selected for inclusion in the manuscript (Fig. 5), showing adequate long-term integration of the graft, absence of scar contracture, and satisfactory cosmetic appearance. At this time, integration and scar quality were independently assessed by three board-certified plastic surgeons using the Vancouver Scar Scale, with two surgeon assigning a score of 2 and the other a score of 3, indicating minimal hypertrophy and erythema, and showing high agreement between evaluators. The patient's mother also reported only minimal alopecia at the scalp donor site and no functional impairment in the affected upper limb.

## 5 Discussion

The current epidemiology of neonatal burn injuries remains poorly defined. A 2022 systematic review identified only 105 published cases over the past four decades, highlighting the rarity of this condition and the limited availability of reliable data. This scarcity underscores the urgent need for countries and healthcare institutions to improve case reporting and data collection. Strengthening epidemiological surveillance would support the development of more effective prevention strategies and offer clearer insights into the global burden, distribution patterns, and management of neonatal burn injuries [1,2].

This case of a third-degree burn in a newborn, caused by a solid heat source in a hospital setting, represents a rare clinical event that presents distinct management challenges. Neonatal burns are infrequently reported in the literature, as most available studies focus on adult and older pediatric populations [1]. Such cases highlight the pressing need for dedicated protocols in neonatal burn care, considering the physiological fragility of this age group and the risks associated with hemodynamic instability—particularly during the early neonatal period [12]. Yu and Park [13] described a full-thickness burn in a 10-day-old neonate resulting from accidental scalding, which was managed with tangential excision and split-thickness skin grafting under general anesthesia. In contrast, Cox et al. (2011) reported the conservative management of a neonate with deep partial-thickness burns using hydrocolloid dressings [22]. While both studies emphasized the importance of intensive care and meticulous fluid management, our case differs in several key aspects: mechanism of injury (contact vs. scald), anatomical location (upper limb vs. lower limb), and approach to wound bed preparation (autolytic debridement vs. surgical excision) [13,14]. Furthermore, unlike the aforementioned reports, our management strategy prioritized minimizing surgical intervention during the acute phase. A staged grafting approach was adopted to reduce systemic stress and allow for progressive wound adaptation.

These distinctions underscore the importance of individualized treatment strategies tailored to burn depth and the unique characteristics of the neonatal patient.

The decision to use hydrogel dressings for autolytic debridement of necrotic tissue was appropriate and well-justified in this context. Compared to more aggressive surgical techniques, autolytic debridement is less invasive and maintains a moist wound environment that promotes endogenous enzymatic breakdown of eschar. This is particularly advantageous in neonates, as it minimizes trauma and reduces the risk of infectious complications. Moreover, this method facilitates optimal wound bed preparation for potential grafting, supporting the recovery of viable tissue without exposing the patient to interventions that could compromise hemodynamic stability [15]. Additionally, the use of cadaveric skin allograft contributes to improved healing rates, temporal coverage, lower pain and reduced scar formation and recurrence. During treatment, the allograft provided temporary coverage, enhanced wound bed preparation, and decreased the risk of infection [4,16,17].

Additionally, the decision to use the scalp as the donor site for skin grafting was based on multiple factors. The two donor site options initially discussed with the parents were the sole of the foot and the scalp, due to their lower incidence of hypertrophic scarring compared to more conventional sites such as the thigh, back, buttocks, or lower leg [5,6]. This choice is supported by Mimoun et al. [5], who concluded that the scalp is an advantageous donor site for thin-skin grafts, offering rapid healing, low complication rates, and favorable cosmetic outcomes. Rotatori et al. (2019) also reported that the thigh carries a significantly higher risk of hypertrophic scarring, whereas the scalp has a markedly lower risk in both the short and long term [23]. Prior to reviewing the evidence, the parents expressed a preference for using the scalp over the sole of the foot, despite being informed of potential risks such as alopecia or folliculitis. Once presented with comparative data showing that the scalp has a slightly lower incidence of persistent hypertrophic scarring (2 %) than the foot (5 %), a shared decision was made between the parents and the surgical team to harvest the split-thickness skin graft from the scalp [6,7].

The main differences between newborns and adults regarding the use of the scalp as a donor site for split-thickness skin grafts lie in healing dynamics and physiological characteristics. Newborns exhibit faster healing due to superior collagen organization during the regenerative phase of wound repair [18,19]. However, they are also more susceptible to proportionally greater blood loss during graft harvesting. As such, meticulous hemostasis must be achieved immediately after excision to prevent systemic complications. This can be effectively managed using tumescent infiltration and epinephrine-impregnated gauze [20]. Furthermore, graft thickness must be carefully controlled in neonates to avoid excessive dermal injury and ensure optimal regeneration, given that the scalp in newborns has an average epidermal thickness of 0.081–0.083 mm and dermal thickness of 0.68–0.72 mm, considering these measurements, derived from the systematic review by Fonseca de-Souza et al. [21], a graft thickness of 0.2 mm was deliberately chosen to include the full epidermis and only the most superficial portion of the dermis. This approach aimed to preserve deeper dermal structures, including the majority of hair follicles and adnexal elements, to promote rapid donor site healing and minimize the risk of alopecia, while ensuring adequate structural integrity for successful graft take.

The management of burn injuries in neonates is hindered by limited data availability and the absence of standardized guidelines, particularly for third-degree burns, which complicates evidence-based clinical decision-making. Uncertainties also persist regarding the optimal type of graft and the ideal timing for its application, given the neonate's ongoing physiological development and heightened vulnerability to metabolic and infectious complications. Moreover, the lack of comparative studies in neonatal burn care prevents the establishment of clinical consensus, underscoring the urgent need for future research to develop population-specific management protocols.

## 6 Conclusions

Burn injuries in neonates represent a significant reconstructive and clinical challenge, largely due to the absence of standardized treatment protocols. This case illustrates the effective use of cadaveric skin allografts for wound bed preparation and demonstrates the scalp as a reliable and anatomically favorable donor site for harvesting autologous partial-thickness skin grafts in neonates. The outcomes emphasize the importance of individualized, physiology-based surgical planning in this vulnerable population. Finally, preventive strategies and rigorous institutional safety protocols remain critical to avoiding iatrogenic burn injuries in healthcare settings.

## Author contribution

**Dr. Paula Andrea Pérez Franco**: Treating plastic surgeon; reviewed the manuscript in multiple stages to ensure clarity.

**Sebastián Murcia**: Treating general practitioner; lead author and corresponding author.

**Mauricio Alfonso Uribe Rodríguez**: Co-author and contributor to manuscript writing.

**Juan Felipe Vera**: Co-author and contributor to manuscript writing.

**Jorge Elías Ochoa**: Co-author and contributor to manuscript writing.

## Ethical approval

This case report was conducted in accordance with all applicable ethical standards for clinical research and publication. Although formal approval from an ethics committee was not required for a single-patient case report, written informed consent was obtained from the patient's legal guardians for publication of the case details and accompanying clinical images.

## Guarantor

Dr. Paula Andrea Pérez Franco and Dr. Sebastián Murcia.

## Research registration number

Not applicable.

## Declaration of Generative AI and AI-assisted technologies in the writing process

All authors declare the use of ChatGPT (OpenAI, GPT-4, March 2024 version) solely to assist in verifying and refining grammar and scientific language during the translation of the manuscript from Spanish to English. The tool was accessed via a cloud-based API and was used exclusively for linguistic enhancement. It did not generate original scientific content, contribute to the study design, or influence the interpretation of results. No fine-tuning or custom parameters beyond the default settings were applied.

## Funding

This research received no specific grant from any funding agency in the public, commercial, or not-for-profit sectors. The sponsors had no role in the design, data collection, analysis, interpretation, writing, or decision to submit this manuscript.

## Conflict of interest statement

The authors declare that they have no known competing financial interests or personal relationships that could have appeared to influence the work reported in this paper. This research did not receive any specific grant from funding agencies in the public, commercial, or not-for-profit sectors.

## References

[1] Muntean A., Stoica I., Enescu D. (2022). Etiology of neonatal burns: a systematic review. Ann. Burns Fire Disasters.

[2] Bresler Richard Mark, Barksdale Elizabeth, Hansen Erik Nels (November/December 2022). Pediatric burn care in the developing world: where are the gaps in research and what can be done?. J. Burn Care Res..

[3] Ugburo A., Fadeyibi I., Mofikoya B., Akanmu O., Temiye E., Kanu O. (2013). Neonatal burns in Lagos, south-western Nigeria: epidemiology and outcome of management. Burns.

[4] Rimdeika R., Bagdonas R. (2005). Major full thickness skin burn injuries in premature neonate twins. Burns.

[5] Mimoun M., Chaouat M., Picovski D., Serroussi D., Smarrito S. (2006). The scalp is an advantageous donor site for thin-skin grafts: a report on 945 harvested samples. Plast. Reconstr. Surg..

[6] Rotatori R. Maxwell, Starr Brian, Peake Mitchell, Fowler Laura, James Laura, Nelson Judy, Dale Elizabeth L. (2019). Prevalence and risk factors for hypertrophic scarring of split thickness autograft donor sites in a pediatric burn population. Burns.

[7] Goldsztein H., Ort S., Roberson J.B., Reinisch J. (2012). Scalp as split thickness skin graft donor site for congenital atresia repair. Laryngoscope.

[8] Wyrzykowski D., Chrzanowska B., Czauderna P. (2015). Ten years later – scalp still a primary donor site in children. Burns.

[9] Bovenberg M.S., Williams P.E., Goldberg L.H. (Mar 1 2024). Assessment of donor site scar outcomes, healing time, and postoperative complications associated with split thickness skin grafts harvested from the hair bearing scalp. Dermatol. Surg..

[10] Neuhaus K., Schiestl C., Adelsberger R., Weibel L., Meuli M., Böttcher-Haberzeth S. (May 2019). Bold to do - bald to be? Outcomes decades after harvesting the scalp in burned children. Burns.

[11] Kerwan A., Al-Jabir A., Mathew G., Sohrabi C., Rashid R., Franchi T., Nicola M., Agha M., Agha R.A. (2025). Revised Surgical CAse REport (SCARE) guideline: an update for the age of Artificial Intelligence. Premier J. Sci..

[12] Saaiq M., Ahmad S., Zaib S. (2013). Neonatal burn injuries: an agony for the newborn as well as the burn care team. Ann. Burns Fire Disasters.

[13] Yu J.E., Park D.H. (Jul-Aug 2010). Full-thickness contact burn from a warming bottle in a newborn. Pediatr. Dermatol..

[14] Mihara K., Shindo H., Ohtani M., Nagasaki K., Nakashima R., Katoh N., Kishimoto S. (Sep 2011). Early depth assessment of local burns by videomicroscopy: 24 h after injury is a critical time point. Burns.

[15] Zamani S., Ehterami A., Vaez A., Naeiji M., Maghsoodifar H., Sadeghi Douki S.A.H., Molaee Eshgh Abad M., Arabpour Z., Baheiraei N., Farahani A., Djalilian A.R., Salehi M. (Jun 30 2025). Natural and synthetic polymers in burn wound healing. J. Biomater. Sci. Polym. Ed..

[16] Mosti G., Mattaliano V., Magliaro A., Picerni P., Bastiani L. (Feb 2020). Cadaveric skin grafts may greatly increase the healing rate of recalcitrant ulcers when used both alone and in combination with split-thickness skin grafts. Dermatol. Surg..

[17] Gaviria Castellanos J.L. (2016). Experiencia en la aplicación de tejidos laminares en pacientes quemados del Hospital Simón Bolívar de Bogotá. Rev. Colomb. Cir. Plást. Reconstr..

[18] Colwell A.S., Longaker M.T., Lorenz H.P. (Sep 1 2003). Fetal wound healing. Front. Biosci..

[19] Visscher M.O., Hu P., Carr A.N., Bascom C.C., Isfort R.J., Creswell K., Adams R., Tiesman J.P., Lammers K., Narendran V. (Oct 19 2021). Newborn infant skin gene expression: remarkable differences versus adults. PLoS One.

[20] Chang L.Y., Yang J.Y., Chuang S.S., Hsiao C.W. (Mar 1998). Use of the scalp as a donor site for large burn wound coverage: review of 150 patients. World J. Surg..

[21] de-Souza I.M.F., Vitral G.L.N., Reis Z.S.N. (Nov 2019). Skin thickness dimensions in histological section measurement during late-fetal and neonatal developmental period: a systematic review. Skin Res. Technol..

[22] Cox S.G., Rode H., Darani A.N., Fitzpatrick-Swallow VL. (2011). Thermal injury within the first 4 months of life. Burns.

[23] Rotatori R.M., Starr B., Peake M., Fowler L., James L., Nelson J., Dale EL. (2019). Prevalence and Risk Factors for Hypertrophic Scarring of Split Thickness Autograft Donor Sites in a Pediatric Burn Population. Burns.

